# A novel near-infrared fluorescent probe for visualization of intracellular hydrogen peroxide

**DOI:** 10.3389/fchem.2022.1025723

**Published:** 2022-10-21

**Authors:** Baoshuai An, Shude Pang, Yanru Zhang, Ningning Wei

**Affiliations:** Department of Pharmacology, School of Pharmacy, Qingdao University Medical College, Qingdao, China

**Keywords:** hydrogen peroxide, fluorescent probe, near-infrared, large Stokes shift, cellular visualization

## Abstract

Hydrogen peroxide (H_2_O_2_) as a crucial reactive oxygen species (ROS) plays a crucial role in redox signaling in physiological and pathological processes of living cells. Its normal production is closely related to signal transduction of living cells. Overproduction of H_2_O_2_ *in vivo* has been proved to be related to many diseases. Some were developed to reveal the roles of H_2_O_2_. However, current fluorescent probes for the detection of H_2_O_2_ are restricted in their short emission wavelengths and small Stokes shifts that significantly decrease the sensitivity of detection and cellular visualization. In this work, a novel fluorescent probe BC-B was designed and synthesized with pinacol phenylboronic acid ester as a recognition group and near-infrared fluorophore BC-OH as a reporter group. BC-B probe exhibits a large Stokes shift (122 nm) and near-infrared emission (672 nm), showing an excellent selectivity and sensitivity in detection of H_2_O_2_ with the limit of 0.003 μmol/L. Confocal fluorescence imaging further demonstrates that BC-B can be used for detecting endogenous H_2_O_2_ in living cells.

## Introduction

H_2_O_2_ is an important reactive oxygen species (ROS) in living systems (Halliwell et al., 2000; Stone and Yang, 2006). Endogenous H_2_O_2_ is mainly produced by NADPH oxidase complexes. Compared with other ROS, H_2_O_2_ has a higher concentration and is more stable *in vivo*. Normal physiological level of H_2_O_2_ plays a vital role in cell damage (Jantas et al., 2020), differentiation (Fujita et al., 2021), apoptosis (Quillet-Mary et al., 1997), iron death (Qi et al., 2021) and oxidative stress (Yang L. et al., 2020). Abnormal level of H_2_O_2_ is implicated in numerous diseases, such as inflammation (McDonald et al., 2020), neurodegenerative diseases (Fukui and Kato, 2021), diabetes (Wang et al., 2021a), ulcerative colitis (Wang et al., 2020) and cancer (Zoumpourlis et al., 1991; Wang et al., 2021b). Therefore, it is of great significance to develop a high effective method with good sensitivity and selectivity to monitor intracellular H_2_O_2_ in biological systems.

Compared with other methods, Fluorescence probe technology used for detection of intracellular H_2_O_2_ has the advantages of non-invasiveness and good biocompatibility (An et al., 2020; Zhang et al., 2020; Luo et al., 2021; Xu et al., 2021; Zheng et al., 2021) over the traditional detection assays including chromatography (Tarvin et al., 2011), mass spectrometry (Cocheme et al., 2011), colorimetry (Zou et al., 2019) and electrochemistry (Liu et al., 2014). Many fluorescent probes for H_2_O_2_ are developed based on small molecular fluorophores such as rhodamine (Gu et al., 2020), coumarin (Du et al., 2010), naphthalimide (Sun et al., 2013), and BODIPY (Purdey et al., 2018; Wei et al., 2021). However, these fluorescent probes usually have short emission wavelength (<650 nm) and small Stokes shift (<100 nm), which limit their detection of H_2_O_2_ in cells or deep tissues. Therefore, it is still necessary to develop a fluorescence probe with large Stokes shift (>100 nm) and near infrared emission (>650 nm) for the detection of intracellular H_2_O_2_.

In this study, we designed and synthesized a novel fluorescent probe BC-B using a malononitrile isophorone derivative (BC-OH) that has large Stokes shift and near infrared emission (Yang X. et al., 2020; Ren et al., 2020; Shu et al., 2020; Shiraishi et al., 2021) as a fluorophore and a phenylboronic acid pinacol ester group as a recognition group. BC-B probe exhibits selective and potent detection of H_2_O_2_ with a large Stokes shift (122 nm) near infrared emission (672 nm), giving rise to desirable imaging of endogenous H_2_O_2_ in living cells.

## Materials and methods

### Materials and instruments

All reagents were purchased from reagent companies and used directly, if not otherwise specified. The water used in the experiment was double distilled water. UV-Vis spectra were measured with Shimadzu UV 2600. Fluorescence spectra were measured by F-7000 fluorescence spectrophotometer. High-resolution mass spectra of compounds were measured by Agilent Q-TOF6510 spectrograph. The NMR spectra of compounds were recorded by Bruker Advance 500 spectrometer. The pH was measured by PHS-3C. Absorbance for MTT assay was determined by TECAN Austria Gmbh A-5082. Confocal imagings were carried out by Nikon A1R MP.

### Synthesis of BC-B

**SCHEME 1:**
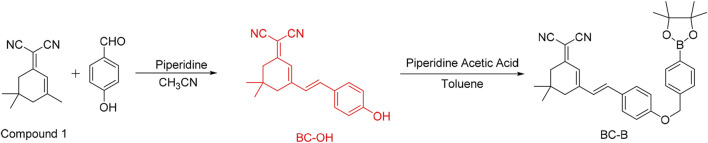
Synthesis of BC-B.

As shown in Scheme 1, compound 1 (2 g, 10.72 mmol) and p-hydroxy benzaldehyde (2 g, 16.3 mmol) were dissolved in 50 ml CH_3_CN before 2 ml acetic acid and 2 ml piperidine were added. The reaction mixture was heated to 120°C and kept in reflux for 12 h under the protection of argon. The reaction mixture was cooled and the solvent was removed under reduced pressure to give the crude product. Finally, the crude product was purified by silica gel column chromatography to give BC-OH as a red solid.

Under the protection of argon, the fluorophore BC-OH (261 mg, 0.9 mmol), 4-bromomethyl phenylboronic acid pinacol ester (88 mg, 0.296 mmol), anhydrous potassium carbonate (369 mg, 0.89 mmol) and NaI (440 mg, 2.93 mmol) were added into 15 ml anhydrous CH_3_CN, and reacted at room temperature for 24 h. After completion of the reaction, the solvent was removed by evaporation under reduced pressure. DCM and saturated sodium chloride solution were used to extract repeatedly. The organic phase was collected and dried by anhydrous sodium sulfate. The filtrate was collected after filtration, and the organic solvent was removed under reduced pressure to give crude product, which was further purified by silica gel column chromatography (DCM: PE = 1: 1, v/v), to obtain orange solid (BC-B) (73 mg, yield 48.74%). ^1^H NMR (500 MHz, DMSO) δ 7.70 (dd, J = 16.2, 8.3 Hz, 4H), 7.47 (d, J = 7.9 Hz, 2H), 7.29 (d, J = 4.6 Hz, 2H), 7.07 (d, J = 8.6 Hz, 2H), 6.85 (s, 1H), 5.22 (s, 2H), 1.31 (s, 14H), 1.03 (s, 6H), 0.89 (s, 2H). ^13^C NMR (126 MHz, DMSO) δ 160.01, 156.90, 140.66, 138.06, 135.05, 130.09, 129.42, 127.90, 127.33, 122.36, 115.80, 113.70, 84.16, 75.83, 69.58, 42.79, 38.66, 32.16, 27.93, 25.15.

### Cytotoxicity test

Cytotoxicity was evaluated in HeLa cells using the MTT assay. HeLa cells were inoculated in culture plate. As adhered to the walls, HeLa cells were incubated with different concentration of BC-B (0, 3, 10, 30 μM) for 24 h. Then, MTT (10 μL) was added and HeLa cells were further cultured for 4 h. Finally, the plate was shaken for about 30 min, and each well was analyzed by the microplate reader (TECAN Austria GmbH A-5082) and detected at the absorbance of 492 nm.

### Confocal imaging of H_2_O_2_ in cells

Confocal imaging experiments were divided into three groups, each of which is parallel for three times (Ex = 561 nm). In the first group, the HeLa cells were incubated with 2 ml BC-B (10 μmol/L) for 110 min. In the second group, HeLa cells were incubated with 2 ml H_2_O_2_ (50 μmol/L) for 30 min and washed with PBS for three times, then incubated with 2 ml BC-B (10 μmol/L) for 110 min. The third group of HeLa cells was incubated with 2 ml H_2_O_2_ (50 μmol/L) for 30 min and washed with PBS for three times, then incubated with 2 ml of NAC (1 mmol/L) (NAC is N-Acetyl-l-cysteine, a remover of endogenous H_2_O_2_) for 1 h, washed with PBS three times, and then incubated with 2 ml of BC-B (10 μmol/L) for 110 min. In endogenous H_2_O_2_ imaging, PMA(1 g/mL)(PMA is Phorbol 12-myristate 13-acetate, a mature H_2_O_2_ inducer) is used to replace H_2_O_2_ added in the second and third groups of exogenous experiments, stimulate cells to produce endogenous hydrogen peroxide, and study its imaging by BC-B. All cells were washed three times with PBS buffer, and the fluorescence images of cells were observed with confocal fluorescence microscope by Nikon A1R MP.

## Results and discussion

### Spectral response of BC-B to H_2_O_2_

**FIGURE 1:**
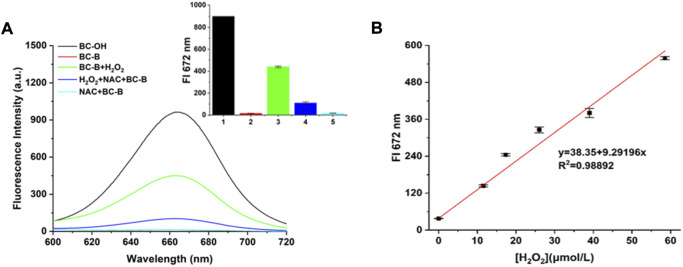
Fluorescence spectra of BC-B probe in response to H_2_O_2_. **(A)** The fluorescence spectra of different groups upon excitation using 550 nm, (Black line: BC-OH fluorophore, Red line: BC-B probe, Green line: BC-B probe + H_2_O_2_, Blue line: BC-B probe + NAC + H_2_O_2_, Blue-green line: BC-B probe + NAC. Inset: fluorescence intensity at 672 nm of different groups. **(B)** The linearity between the fluorescence intensity of BC-B probe and the concentration of H_2_O_2_ from 0 to 60 μmol/L, Ex: 550 nm.

**SCHEME 2:**
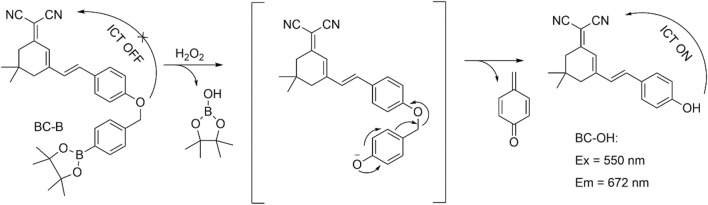
The mechanism of BC-B-specific detection of H_2_O_2_.

At first, the fluorescence response of BC-B to H_2_O_2_ was measured in PBS solution at pH7.4 with excitation of 550 nm. As shown in Figure 1A, the BC-B did not emit fluorescence at 672 nm, suggesting that the phenylboronic acid pinacol ester group of the probe BC-B quenched the fluorescence of BC-OH fluorophore. While H_2_O_2_ (50 μmol/L) was added into the BC-B (10 μmol/L) system, the system emitted strong fluorescence at 672 nm with excitation of 550 nm. The emission wavelength of BC-B with H_2_O_2_ is the same to that of the fluorophore BC-OH. The results suggested that the probe BC-B can react with H_2_O_2_ to release BC-OH fluorophore, showing strong fluorescence. The H_2_O_2_ (50 μmol/L) solution pretreated with reactive oxygen scavenger NAC (1 mmol/L) for 5 min was added into BC-B solution (10 μmol/L) and incubated for 110 min, the fluorescence intensity of the system at 672 nm is significantly weaker than that of probe BC-B with H_2_O_2_ group, but stronger than that of the free probe BC-B group. When the probe BC-B was incubated with NAC (1 mmol/L) for 110 min, the fluorescence intensity was the same to that of BC-B. As the probe BC-B reacted with H_2_O_2_, the UV absorption peak appeared at 550 nm. The UV absorption spectral is similar to that of BC-OH (Supplementary Figure S1). These data further illustrated that H_2_O_2_ made BC-B probe release BC-OH fluorophore and showing strong fluorescence, we used HPLC to examine the mechanism of the probe BC-B (Scheme 2 and Supplementary Figure S2).

The fluorescence intensity of BC-B system at 672 nm increased with the concentration of H_2_O_2_, and a good linearity was built between the fluorescence intensity of BC-B probe and the concentration of H_2_O_2_ (y = 38.35 + 9.29196x, *R* ^2^ = 0.98892) (Supplementary Figure S3A). The limit of detection was calculated to be 3 nmol/L according to the equation LOD = 3σ/k. These results show BC-B has high sensitivity to H_2_O_2_ and can quantitatively detect H_2_O_2_ *in vitro*.

### Time- and pH-dependent effect of BC-B probe on detecting H_2_O_2_

**FIGURE 2:**
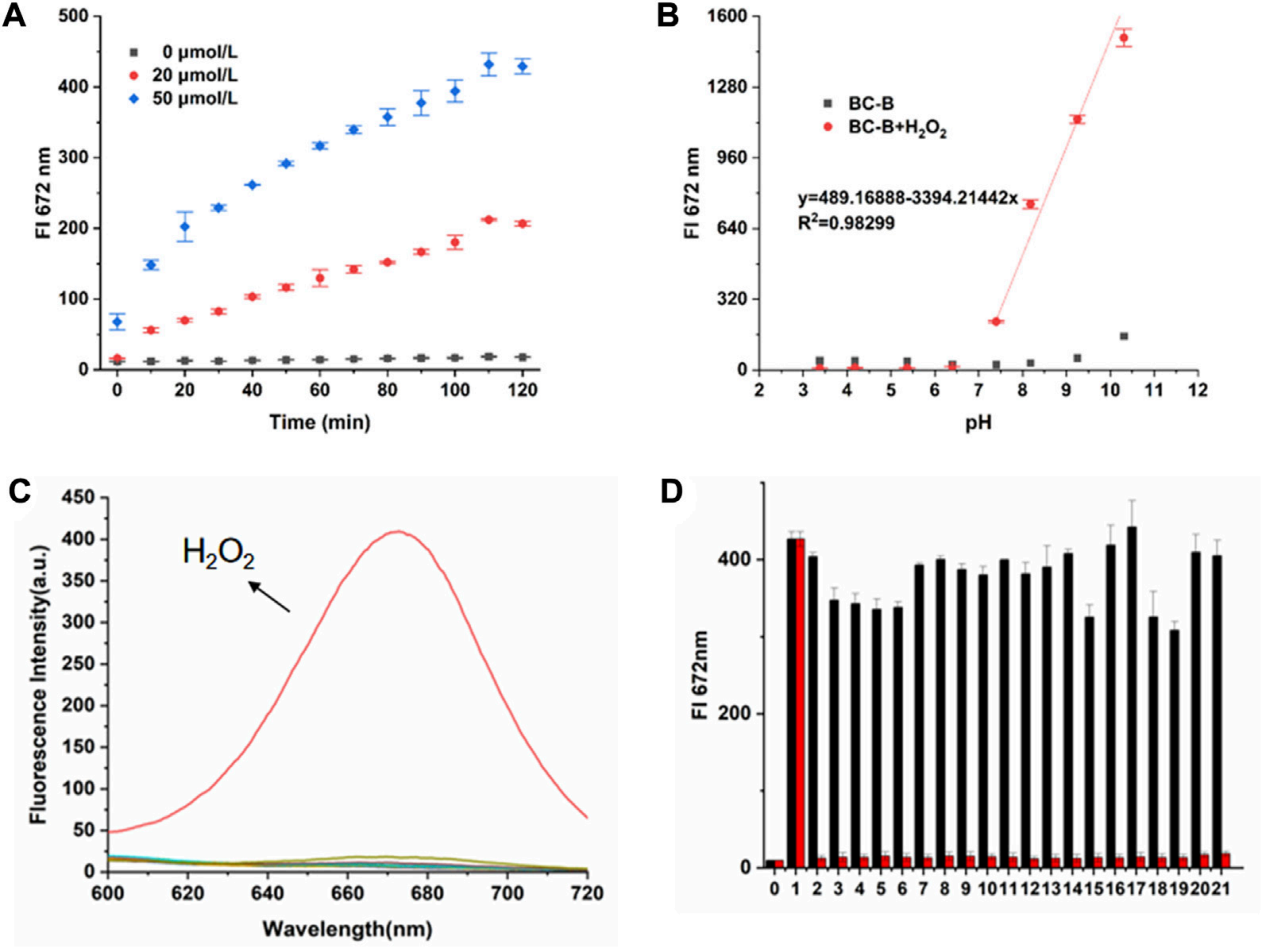
Spectral response of BC-B to H_2_O_2_. **(A)** Changes of fluorescence intensity at 672 nm (Ex = 550 nm) during 0–120 min incubation in BC-B system or BC-B system containing H_2_O_2_ (20 μmol/L, 50 μmol/L). **(B)** The changes of fluorescence intensity at 672 nm in BC-B system or BC-B system containing H_2_O_2_ after incubation at different pH for 110 min. **(C)** The fluorescence spectra of the BC-B system containing H_2_O_2_ or other analytes after incubation for 110 min (Ex = 550 nm). **(D)** Selectivity of probe BC-B towards various analytes (Ex = 550 nm). Red: BC-B + H_2_O_2_ or other analytes; Black: BC-B + H_2_O_2_ and other analytes. (0. BC-B, 1. BC-B + H_2_O_2_, 2. NaClO, 3. FeCl_3_, 4. MgCl_2_, 5. NaBr, 6. CaCl_2_, 7. NH_4_Cl, 8. KI, 9. MgSO_4_, 10. NaHCO_3_, 11. L-Pro, 12. L-Arg, 13. β-Ala, 14. glucose, 15. L-Cys, 16. NaNO_2_, 17. Na_2_S, 18. NAC, 19. GSH, 20. SNP, 21. C_4_H_10_O_2_).

First, the time-dependence of BC-B for detecting H_2_O_2_ was evaluated through measuring the fluorescence of BC-B after incubated with or without H_2_O_2_ for different time. As shown in Figure 2A and Supplementary Figure S3B, the free probe BC-B shows a little fluorescence in 120 min, indicating that BC-B probe is very stable in PBS solution. As H_2_O_2_ was added into the solution of BC-B, the fluorescence intensity (672 nm) of the system increased gradually during the incubation time of 0–110 min, and reached the maximum value at 110 min. Then, we measured the fluorescence changes of BC-B in presence or absence of H_2_O_2_ at different pH ranging from 3.4 to 10.5. As shown in Figure 2B and Supplementary Figure S3C, the fluorescence intensity of BC-B (10 μmol/L) at 672 nm was relatively stable with excitation at 550 nm. After incubation of BC-B with H_2_O_2_ (20 μmol/L) for 110 min, there was no fluorescence response under acidic conditions, while a good fluorescence response under alkaline conditions (pH = 7.4), this indicated that the BC-B has an ability to detect H_2_O_2_ under alkaline conditions.

### Selectivity and anti-interference capacity of BC-B

The selectivity of analytes is an important parameter to evaluate property of fluorescent probes. It can be seen from Figure 2C that H_2_O_2_ induces a significant increase in the fluorescence intensity of BC-B, while the fluorescence intensity of other active species changes slightly, which indicates that the BC-B can specifically respond to H_2_O_2_. Then, add other active species to the solution of BC-B and H_2_O_2_ for interference test. Compared with the BC-B probe using H_2_O_2_, the fluorescence of these test samples has almost no change, showing good anti-interference ability. These results revealed that BC-B has excellent selectivity for H_2_O_2_ and has potential application in visualization of H_2_O_2_ in complex cell environment (Figure 2D **)**.

### Confocal imaging of HeLa cells for detection of H_2_O_2_ level by BC-B

**FIGURE 3:**
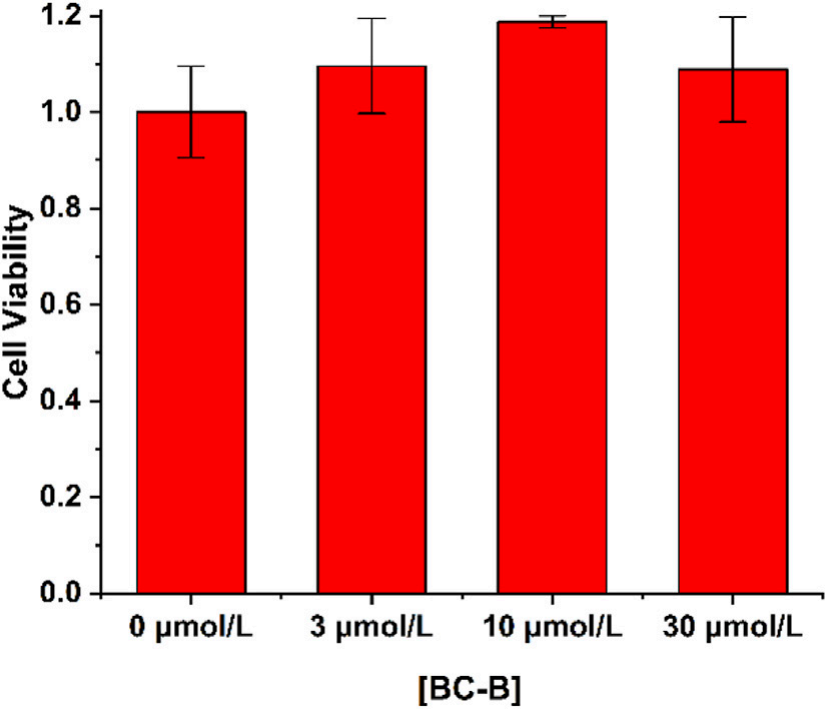
The cytotoxicity of BC-B (0–30 μmol/L) against HeLa cells was analyzed by using MTT assay.

Cell survival percentage was calculated by MTT assay. As shown in Figure 3, the survival percentage of the 0 μmol/L BC-B was considered equal to 1 and the survival percentage of other samples was calculated according to the 0 μmol/L BC-B. High viability values of HeLa cells were obtained when treated with 30 μmol/L BC-B. Cell viability values measured by the MTT assay demonstrate that BC-B has a little cytotoxicity, and has the potential to be used to visualize H_2_O_2_ in HeLa.

**FIGURE 4:**
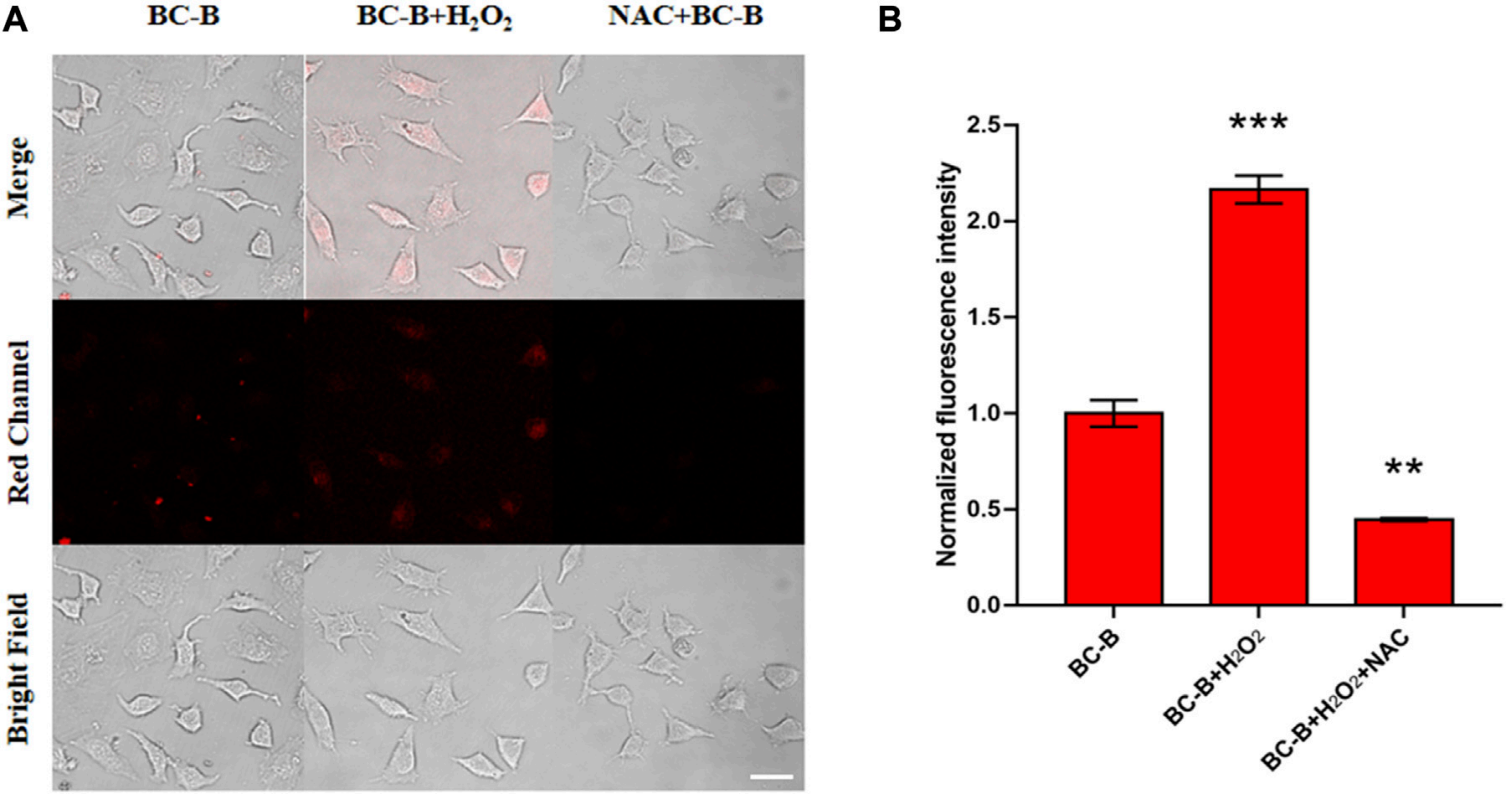
Fluorescence images of exogenous H_2_O_2_ in HeLa cells. **(A)** First column (Control group): the cells were treated with BC-B (10 μM) for 110 min. Second column (H_2_O_2_ group): the cells were treated with H_2_O_2_ (50 μM) for 30 min and then BC-B (10 μM) for 110 min. Third column (BC-B + NAC group): the cells were incubated with H_2_O_2_ (50 μM) for 30 min, NAC (1 mM) for 1 h, and then BC-B (10 μM) for 110 min. **(B)** Bar graph representing the normalized fluorescence intensity of the three groups in panel A, Ex: 561 nm, Em: 663–738 nm; scale bars: 50 μm.

Based on the excellent sensitivity, selectivity and biocompatibility of BC-B for H_2_O_2_, the ability of BC-B to visualize intracellular H_2_O_2_ was tested. As shown in Figure 4, compared with the BC-B group, the fluorescence intensity of the red channel in the BC-B + H_2_O_2_ group was significantly enhanced, this result indicates that BC-B can be used to visualize exogenous H_2_O_2_ in HeLa cells. After treating H_2_O_2_ incubated HeLa cells with active oxygen scavenger NAC, the fluorescence intensity of HeLa cells (NAC + BC-B group) incubated by BC-B was significantly weak comparing to that of BC-B group, which indicates that BC-B can be used to visualize endogenous H_2_O_2_ in HeLa cells.

**FIGURE 5:**
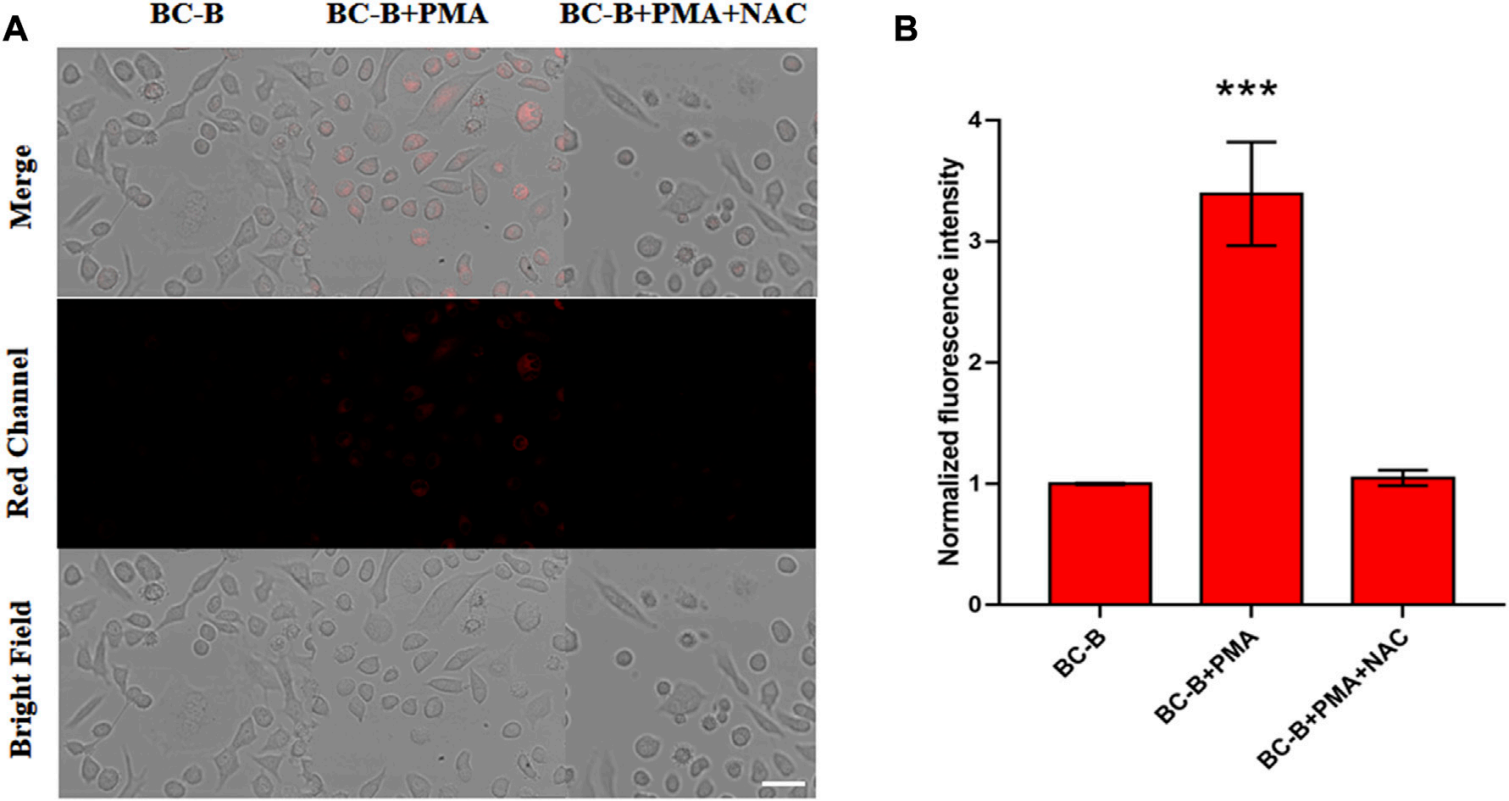
Fluorescence images of endogenous H_2_O_2_ in HeLa cells. **(A)** First column (control): the cells were treated with BC-B (10 μM) for 110 min. Second column (PMA group): the cells were treated with PMA (1 μg/ml) for 30 min and then BC-B (10 μM) for 110 min. Third column (PMA + NAC group): the cells were incubated with PMA (1 μg/ml) for 30 min, NAC (1 mM) for 1 h, and then BC-B (10 μM) for 110 min. **(B)** Bar graph representing the normalized fluorescence intensity of the three groups in panel A, Ex: 561 nm, Em: 663–738 nm; scale bars: 50 μm.

In order to detect endogenous H_2_O_2_, PMA is used to stimulate the excessive production of H_2_O_2_ in cells. As expected, PMA-treated cells showed brighter fluorescence than untreated cells (Figure 5A). In addition, as shown in the third column of Figure 5A, the bright fluorescence is inhibited after adding NAC. The normalized fluorescence intensity shows the fluorescence change of the three groups (Figure 5B). Therefore, BC-B can image the endogenous H_2_O_2_ produced by living cells.

## Conclusion

In this study, a near-infrared fluorescent probe BC-B was designed and synthesized for detection of intracellular H_2_O_2_ level. The BC-B probe composed of BC-OH as a fluorophore and a phenylboronic acid pinacol ester as a recognition group is featured with a large Stokes shift, low toxicity and high selectivity. Confocal imaging revealed that BC-B probe was capable of detecting and visualizing endogenous or exogenous levels of H_2_O_2_ in living HeLa cells.

## Data Availability

The original contributions presented in the study are included in the article/Supplementary Material, further inquiries can be directed to the corresponding author.
