# Supplementary material for: A novel near-infrared fluorescent probe for visualization of intracellular hydrogen peroxide
Source: Front Chem. 2022 Oct 21;10:1025723. doi: 10.3389/fchem.2022.1025723 (PMC9634107; doi:10.3389/fchem.2022.1025723)
Supplement: Supplementary file 1 [file DataSheet1.docx]

Supplementary Material


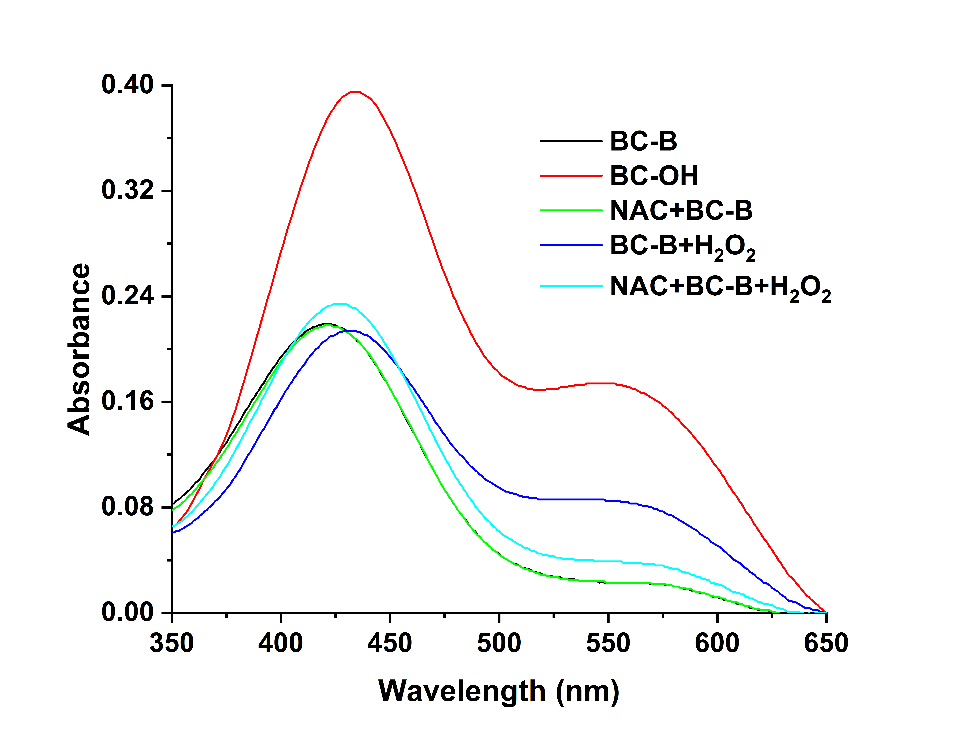


**Supplementary Figure 1.** UV-vis spectra confirmed the specific reaction of BC-B and H_2_O_2_.

**^
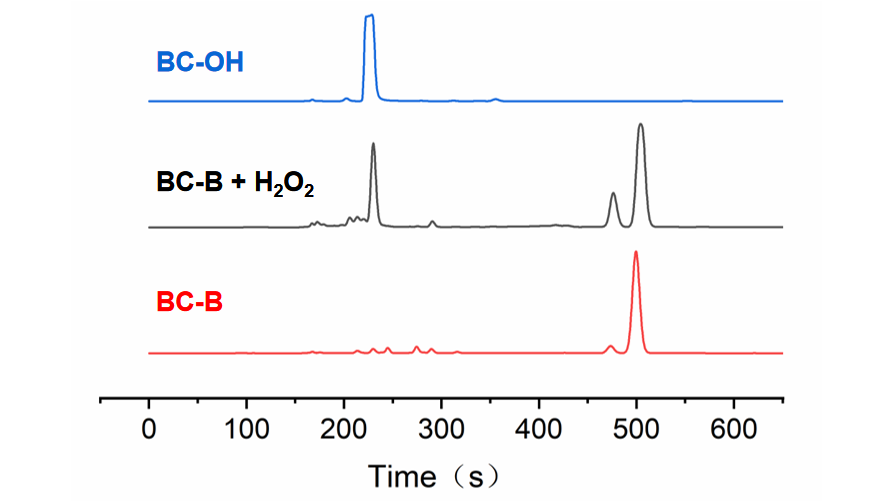
^**

**Supplementary Figure 2.** HPLC chromatograms of BC-B, BC-B with H_2_O_2_, and BC-OH. Mobile phase: acetonitrile/H_2_O = 93/7 (v/v).


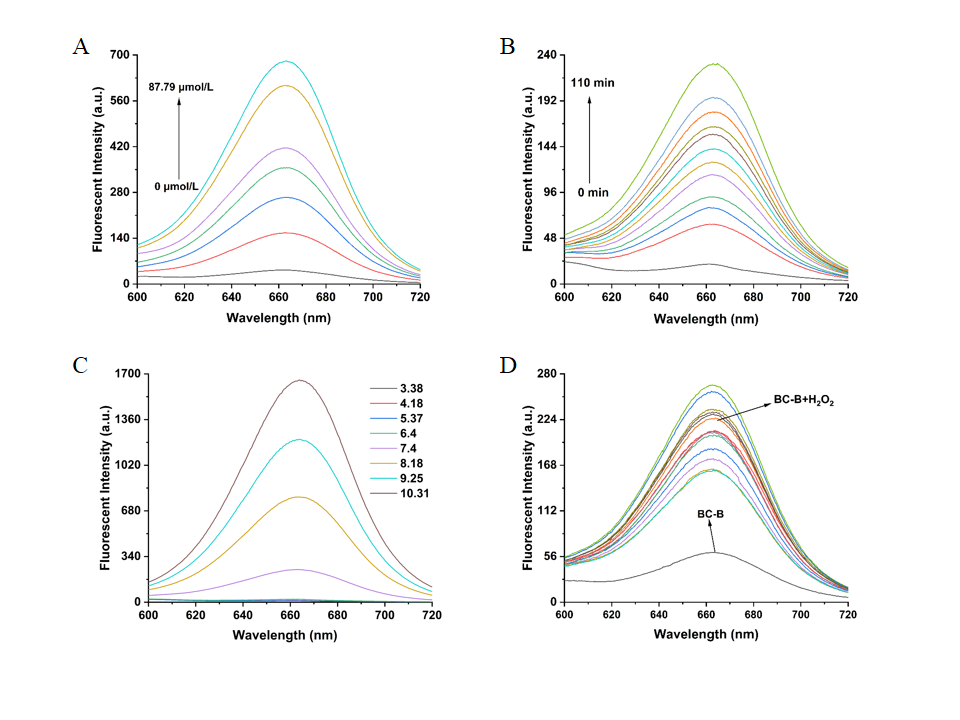


**Supplementary Figure 3. (A)**Fluorescence spectra of the BC-B system with different concentrations of H_2_O_2_ (Ex = 550 nm) were incubated for 110 min. **(B)** Fluorescence spectra of BC-B system incubated with H_2_O_2_ (20 μmol/L) for 0-110 min (Ex = 550 nm). **(C)** Fluorescence spectra of BC-B systems containing H_2_O_2_ after incubation for 110 min at different pH values (Ex = 550 nm). **(D)** After incubation for 110 min, the fluorescence spectra of the BC-B system containing H_2_O_2_ and other analytes (Ex = 550 nm).


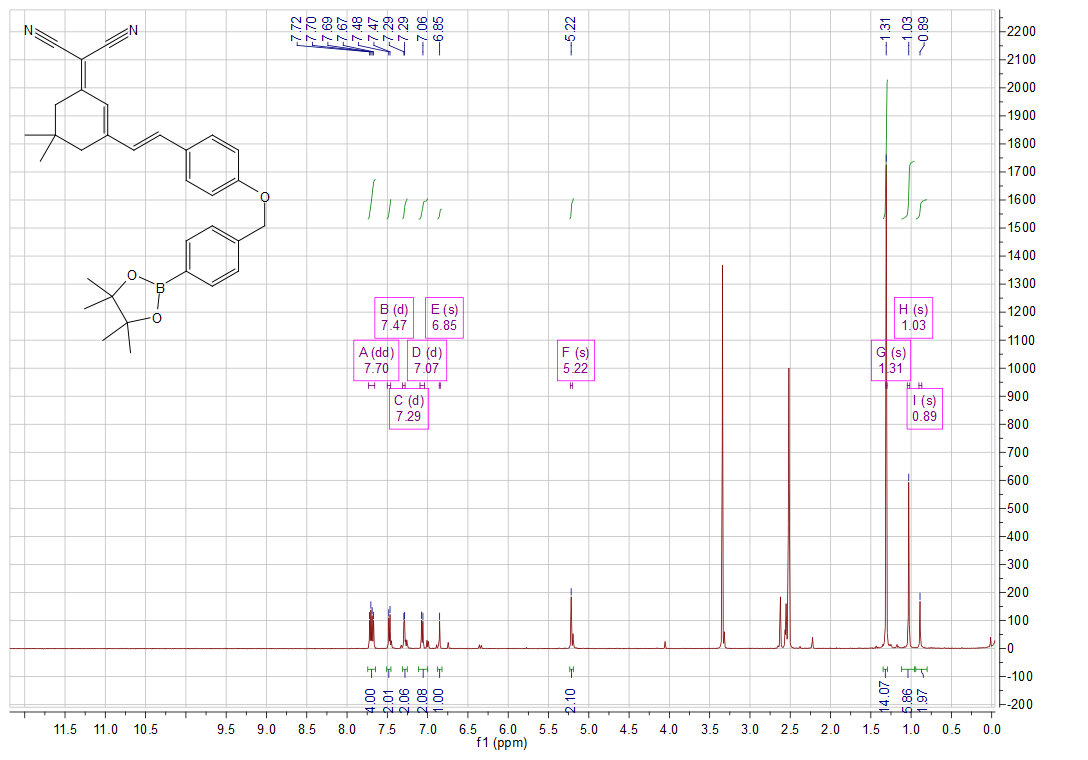


**Supplementary Figure 4.** ^1^H NMR spectra of BC-B in DMSO-d_6_.

**
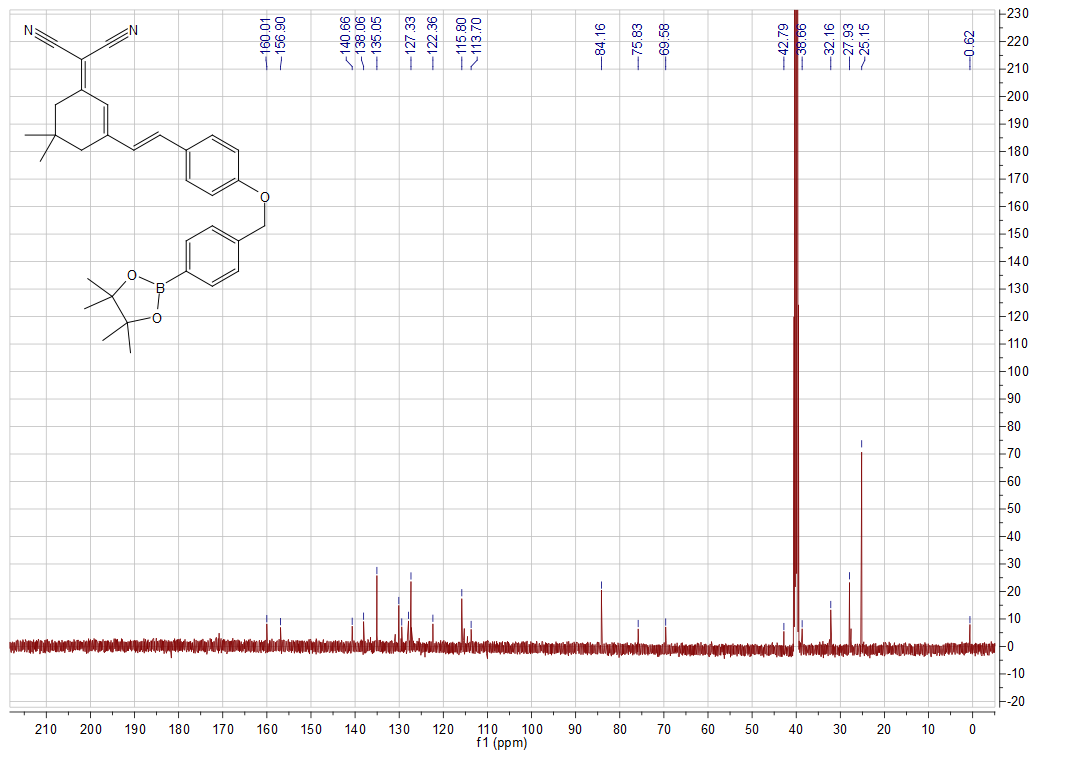
**

**Supplementary Figure 5.** ^13^C NMR spectra of BC-B in DMSO-d_6_.


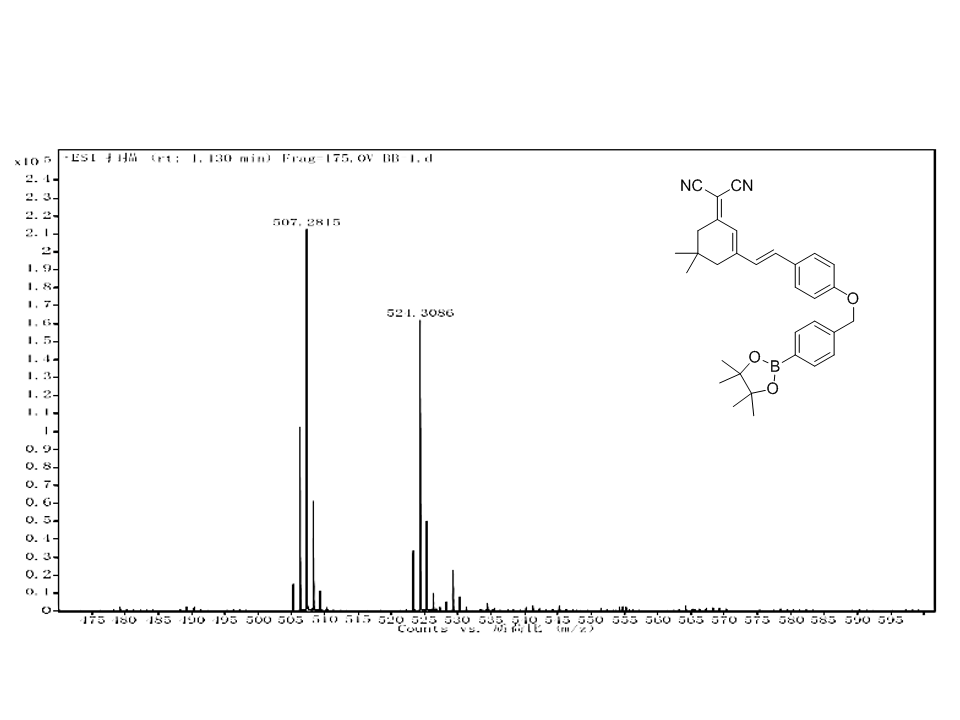


**Supplementary Figure 6.** Mass spectra of BC-B.


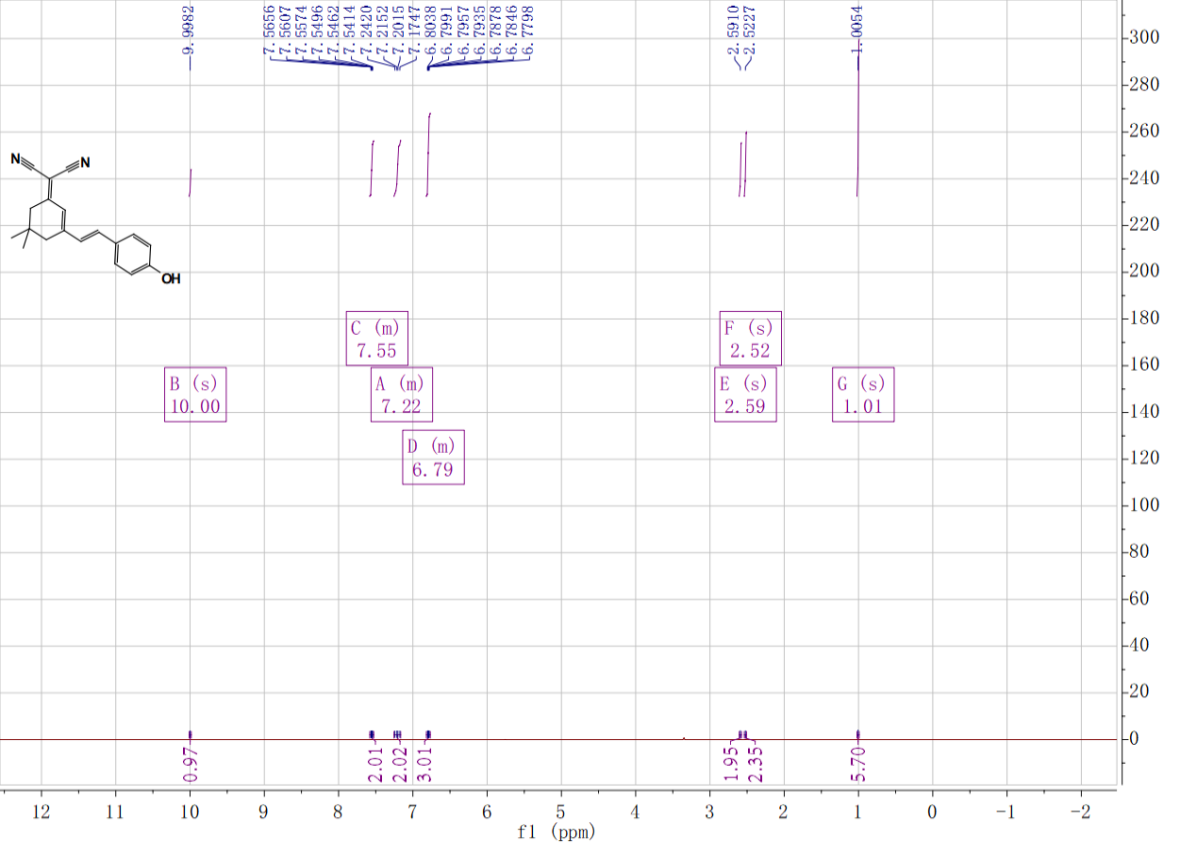


**Supplementary Figure 7.** ^1^H NMR spectra of BC-OH in DMSO-d_6_.


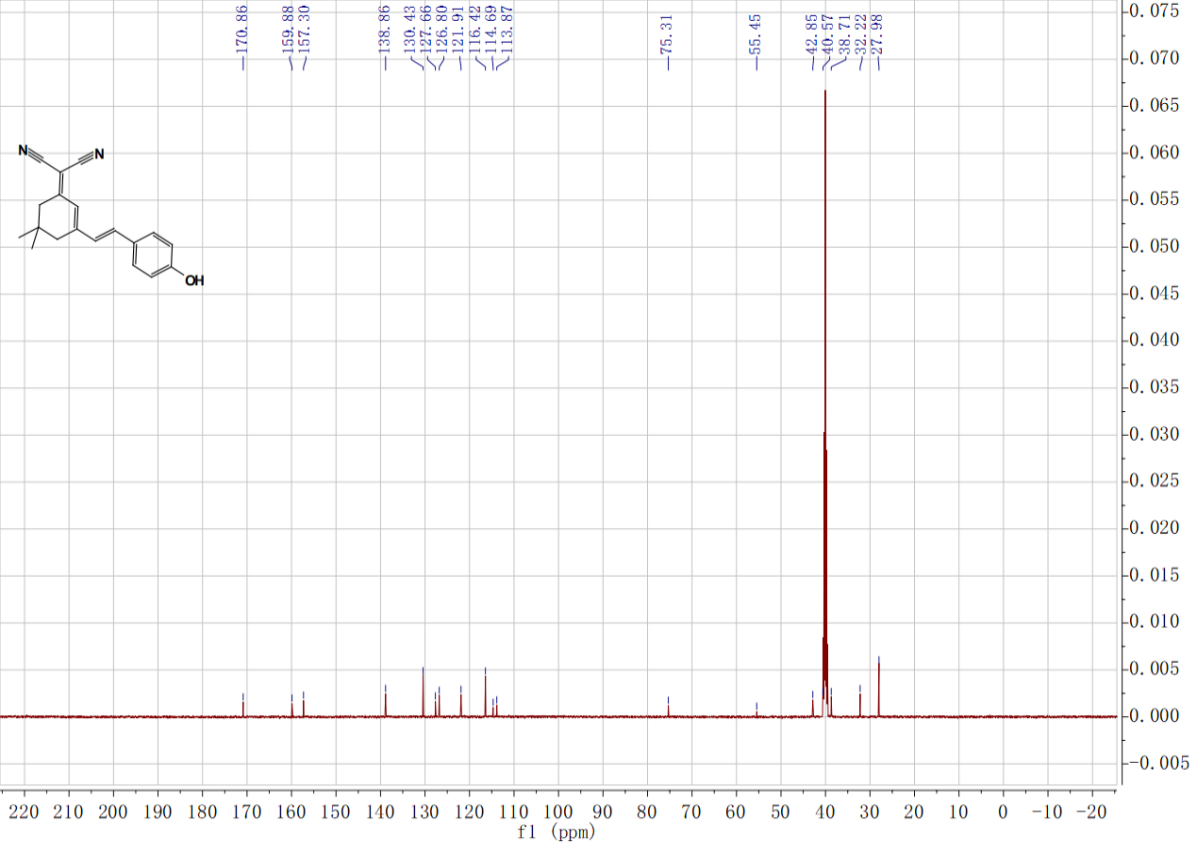


**Supplementary Figure 8.** ^13^C NMR spectra of BC-OH in DMSO-d_6_.


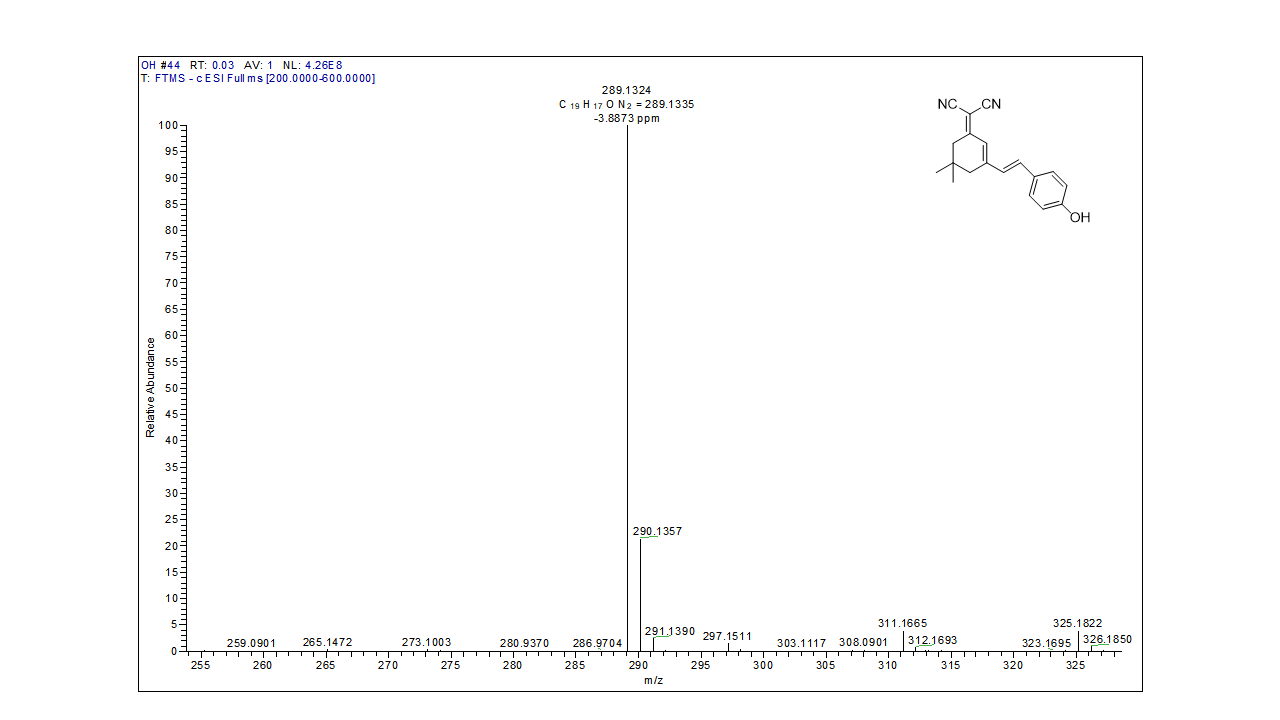


**Supplementary Figure 9.** Mass spectra of BC-OH.
